# Three-Dimensional High-Frequency Ultrasonography for Early Detection and Characterization of Embryo Implantation Site Development in the Mouse

**DOI:** 10.1371/journal.pone.0169312

**Published:** 2017-01-03

**Authors:** Mary C. Peavey, Corey L. Reynolds, Maria M. Szwarc, William E. Gibbons, Cecilia T. Valdes, Francesco J. DeMayo, John P. Lydon

**Affiliations:** 1 Department of Molecular and Cellular Biology, Baylor College of Medicine, Houston, Texas, United States of America; 2 Department of Obstetrics and Gynecology, Division of Reproductive Endocrinology and Infertility, Baylor College of Medicine, Houston, Texas, United States of America; 3 Mouse Phenotyping Core, Baylor College of Medicine, Houston, Texas, United States of America; 4 Reproductive and Developmental Biology Laboratory, National Institute of Environmental Health Sciences, Research Triangle Park, North Carolina. United States of America; Konkuk University, REPUBLIC OF KOREA

## Abstract

Ultrasonography is a powerful tool to non-invasively monitor in real time the development of the human fetus *in utero*. Although genetically engineered mice have served as valuable *in vivo* models to study both embryo implantation and pregnancy progression, such studies usually require sacrifice of parous mice for subsequent phenotypic analysis. To address this issue, we used three-dimensional (3-D) reconstruction *in silico* of high-frequency ultrasound (HFUS) imaging data for early detection and characterization of murine embryo implantation sites and their development *in utero*. With HFUS imaging followed by 3-D reconstruction, we were able to precisely quantify embryo implantation site number and embryonic developmental progression in pregnant C57BL6J/129S mice from as early as 5.5 days post coitus (d.p.c.) through to 9.5 d.p.c. using a VisualSonics Vevo 2100 (MS550S) transducer. In addition to measurements of implantation site number, location, volume and spacing, embryo viability *via* cardiac activity monitoring was also achieved. A total of 12 dams were imaged with HFUS with approximately 100 embryos examined per embryonic day. For the post-implantation period (5.5 to 8.5 d.p.c.), 3-D reconstruction of the gravid uterus in mesh or solid overlay format enabled visual representation *in silico* of implantation site location, number, spacing distances, and site volume within each uterine horn. Therefore, this short technical report describes the feasibility of using 3-D HFUS imaging for early detection and analysis of post-implantation events in the pregnant mouse with the ability to longitudinally monitor the development of these early pregnancy events in a non-invasive manner. As genetically engineered mice continue to be used to characterize female reproductive phenotypes, we believe this reliable and non-invasive method to detect, quantify, and characterize early implantation events will prove to be an invaluable investigative tool for the study of female infertility and subfertility phenotypes based on a defective uterus.

## Introduction

The underlying mechanisms of recurrent early pregnancy loss and infertility are varied, from intrinsic embryonic abnormalities to defects in endometrial receptivity [1–3]. Because of their genetic tractability, murine models represent valuable tools for studies on early implantation and pregnancy progression. Along with their shorter gestational times, the ability to perform large-scale animal studies ensures that the mouse will serve as an important surrogate for human reproductive research in the foreseeable future [4]. However, the majority of murine pregnancy studies still require that numerous animals are euthanized at sequential gestational days to detect and analyze implantation site number, location, spacing and size [5–9] prohibiting the researcher from performing longitudinal studies on the same animal.

In the clinic, ultrasound is an irreplaceable tool used to non-invasively monitor fetal viability and development [10–12]. Similarly, high-frequency ultrasound (HFUS) recently applied to the mouse has been used as an approach to accurately monitor pregnancy progression [13–15]. More recently, advances in ultrasound technology now allow the generation of three-dimensional (3-D) data for visual reconstruction of murine organs and their pathologies [16–18]. With this refined imaging approach, serial imaging reduces inter-animal variability, enhances the power to detect small volume changes, and allows for monitoring of the severity of a pathology and the efficacy of a therapeutic intervention[18]. Most notably, this technology has been used to non-invasively monitor malignancy development in mouse cancer models [16, 18, 19]. However, 3-D HFUS imaging has yet to be applied to monitor the dynamic growth and expansion of embryo implantation events in the mouse uterus.

This short technical report describes the use of non-invasive 2-D HFUS imaging data to generate 3-D visual reconstructions in mesh and solid overlay formats of the murine gravid uterus; these studies also include the detection of early embryonic implantation events and the monitoring of fetal viability without the need for pregnancy termination. Novel 3-D reconstruction modeling *in silico* also provided unique real time data on the rapid structural changes that occur in the uterus and embryo with pregnancy progression. Therefore, we demonstrate the feasibility of using non-invasive 3-D HFUS imaging to detect and monitor pregnancy events as early as 5.5 d.p.c. in the mouse without premature pregnancy termination.

## Materials and Methods

### Ethics Statement

All mouse studies were conducted in accordance with the Guide for the Care and Use of Laboratory Animals published by the National Institutes of Health and animal protocols approved by the Institutional Animal Care and Use Committee (IACUC) of Baylor College of Medicine under protocol number AN-4203.

### Animals

Mice were maintained in a recurrent photocycle of 12h on-off in temperature controlled (22°C ± 2°C) rooms within an AAALAC accredited *vivarium* managed by the Center for Comparative Medicine at Baylor College of Medicine. Mice received a standard diet of irradiated Tekland global soy protein-free extruded food pellets (Harlan Laboratories Inc., Indianapolis, IN) and fresh water *ad libitum*. Animal handling and procedures were performed following the guidelines detailed in the Guide for the Care and Use of Laboratory Animals (National Research Council (Eighth Edition 2011).

### Mouse Breeding

Virgin C57BL6 female mice (6–8 weeks old) were mated with proven fertile isogenic males. Breeding pairs were placed together at 1600 hours and separated by 0700 hours the following morning, which was designated 0.5 day post-coitum (d.p.c) or embryonic day 0.5 (E0.5). Dams were separated from males after one night; pregnant dams underwent imaging at the indicated gestational days through to 9.5 d.p.c.

### High Frequency Ultrasound Imaging

Beginning at 5.5 d.p.c., HFUS imaging was used to detect embryo implantation sites and measure subsequent fetal viability and development. Dams were anesthetized with 3% isoflurane (IsoThesia™, Henry Schein, Melville, NY) and placed on an electric heating pad to prevent hypothermia; abdominal hair was removed with depilatory cream (Nair^TM^ Church & Dwight Co. Trenton, NJ). Dams were positioned supine to monitor heart rate and body temperature. Implantation site detection and location within each uterine horn along with measurements of embryo viability, and fetal size progression were visualized transabdominally using the VisualSonics Vevo® 2100 Imaging System with 550s scan head (FUJIFILM VisualSonics Inc., Toronto, ON) at 55 megahertz. Fetal viability was documented by cardiac activity *via* M-mode Doppler beginning at 9.5 d.p.c. The 3-D Mode was used for advanced data acquisition and analysis, with virtual sections obtained in all directions (x-, y-, z- and other plane variations). Scan distance was set at 10.2mm, with a step size of 0.152 mm and a total of 67 frames were captured per 3-D scan. Each ultrasound was completed within 10–15 minutes; dams were monitored until fully recovered from anesthesia.

### Monitoring Fetal Viability and Growth

Dams were monitored with HFUS on each gestational day, beginning at 5.5 d.p.c. Measurements included: endometrial thickness on morning of plug (0.5 d.p.c.) and embryo implantation site number, location, spacing distance between implantation sites and individual implantation site volume; fetal cardiac activity was confirmed on 9.5 d.p.c. The 3-D image reconstructions were generated for early post-implantation sites and uterine horns from 5.5 d.p.c. through 8.5 d.p.c.

### Image and Data Analysis

After still frame 2-D images were obtained in real time at each gestational age, the images were analyzed and measurements (implantation location, number, and spacing distance) manually determined and calculated using the Vevo® Lab software studio measurement package. The Vevo® Lab software was then used to construct the scans into a 3-D image, which allowed for accurate implantation site volume measurement and image sculpting creating a visual representation of the pregnant uterus. Image sculpting was performed using parallel segmentation with a step size of 0.08mm for contouring and then subsequently depicted with either a mesh or solid overlay. This allowed for a visual representation of the structure of the pregnant uterine horns in 3-D, identifying the number and location of pregnancy sites. A subset of 6 pregnancies (44 pregnancy sites) from 5.5 to 8.5 d.p.c underwent 3-D reconstruction calculations for the creation of a standardized implantation site volume growth curve. A subset of mice which underwent both 2-D and 3-D ultrasound imaging of bilateral pregnant uterine horns were followed by immediate gross tissue dissection. The mice were euthanized by cervical disarticulation while under surgical plane of anesthesia and CO2 euthanasia was conducted via automated CO2 euthanasia chambers (EUTHANEX). The reproductive tract was then excised to visually confirm the ultrasound imaging data with the gross tissue specimen.

### Statistical Analysis

The arithmetic average values for total implantation site locations (n = 98), distance between the implantation sites (n = 49) and volume measurements (n = 44) were calculated and represented as the mean. The amount of variation from the mean value was represented by the standard deviation of the data set.

## Results

**Fig 1:**
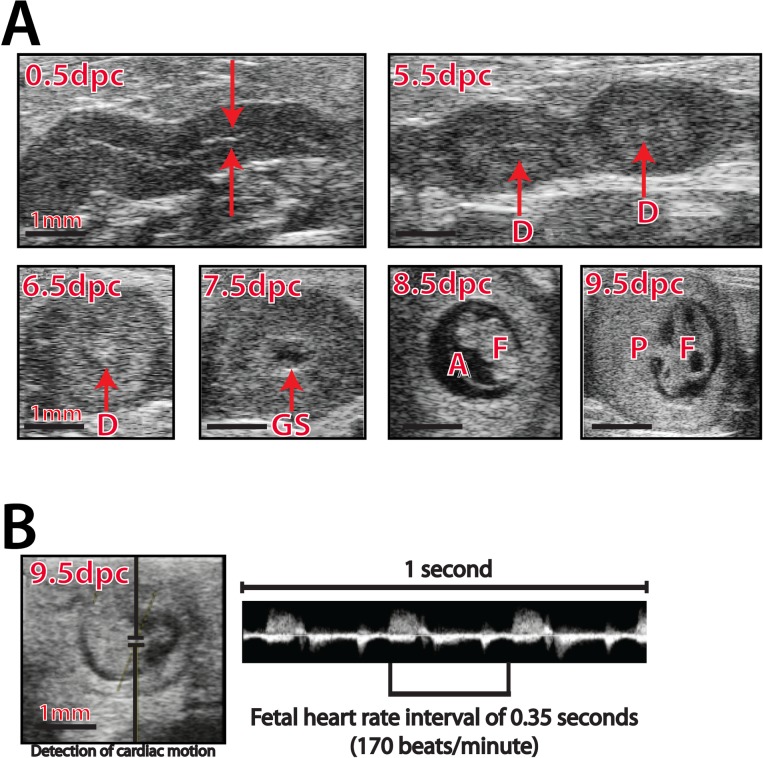
High Frequency Ultrasound detection of implantation site development and monitoring of fetal growth throughout gestation. (**A**) The morning after overnight mating was considered 0.5 d.p.c. The endometrial lining, developing implantation sites and fetal structures were visualized from 0.5 d.p.c. to 9.5 d.p.c. Implantation site number and location, decidualized endometrium (D) on 6.5 d.p.c, gestational sac (GS) on 7.5 d.p.c, fetus (F), amniotic membrane (A) on 8.5 d.p.c, and placenta (P) and fetus (F) on 9.5 d.p.c. are indicated by red arrows. (**B**) At 9.5 dpc, pulse wave Doppler placed over the cardiac ventricles allows for recording of fetal viability as measured by cardiac activity and heart rate; a typical pulse waveform pattern is shown.

A total of 12 dams were monitored with HFUS on each gestational day (5.5 d.p.c to 9.5 d.p.c), with an average of 98 embryos examined per embryonic day as shown in **Fig 1A**. The 2-D measurements of endometrial thickness were obtained at 0.5 d.p.c. Beginning as early as one day after implantation (5.5 d.p.c), structures such as the echogenic decidual reaction, number of implantation sites, location, and embryo spacing could be visualized. With continued development (6.5 to 7.5 d.p.c), the decidualization site and gestational sac were visible; a fetus with amniotic membrane could be measured by 8.5 d.p.c. Structures such as umbilical cord, cardiac activity, and placenta were visible and reliably identified by 9.5 d.p.c. As early as 9.5 d.p.c, pulse wave Doppler was able to detect both umbilical cord and cardiac blood flow (**Fig 1B**), allowing for identification of fetal cardiac activity (heart beat), heart rate, and permit determination of timing of a fetal demise, as would be represented by absence of a heartbeat.

**Fig 2:**
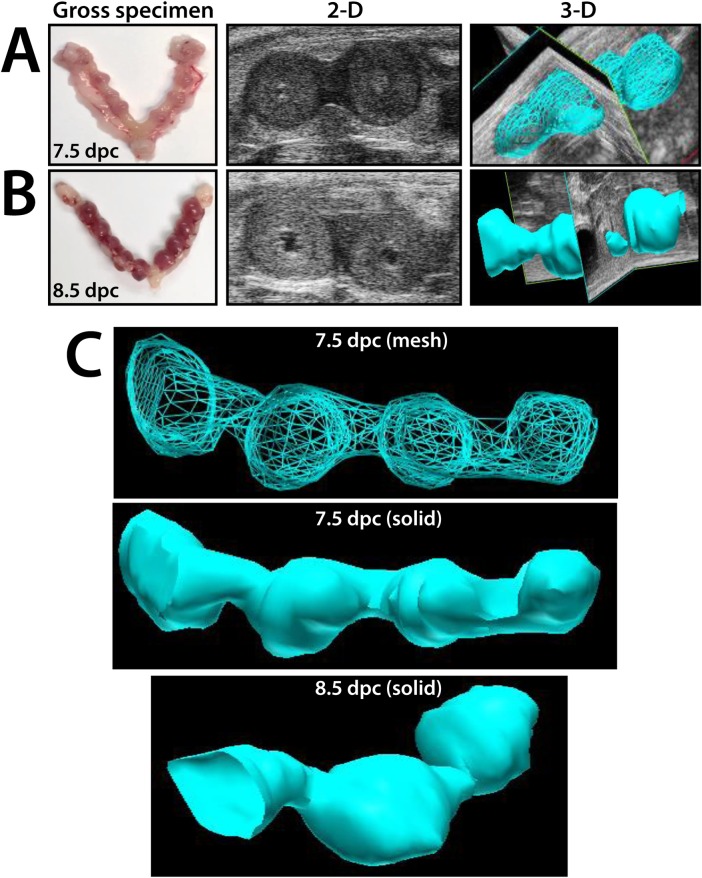
The three-dimensional reconstruction of the mouse uterus during pregnancy. (**A**) At 7.5 d.p.c, both 2-D and 3-D high frequency ultrasound images were obtained of the pregnancy implantation sites and correlated to the gross specimen. (**B**) At 8.5 d.p.c., 2-D and 3-D high frequency ultrasound images were obtained of the pregnancy implantation sites and correlated to the gross specimen. (**C**) The 3-D images were represented without the surrounding intra-abdominal contextual tissue. The four implantation sites of one horn are visualized with both mesh overlay and solid overlay at 7.5 d.p.c.; a 3-D solid overlay representation of a uterus from a 8.5 d.p.c mouse is also shown.

**Fig 3:**
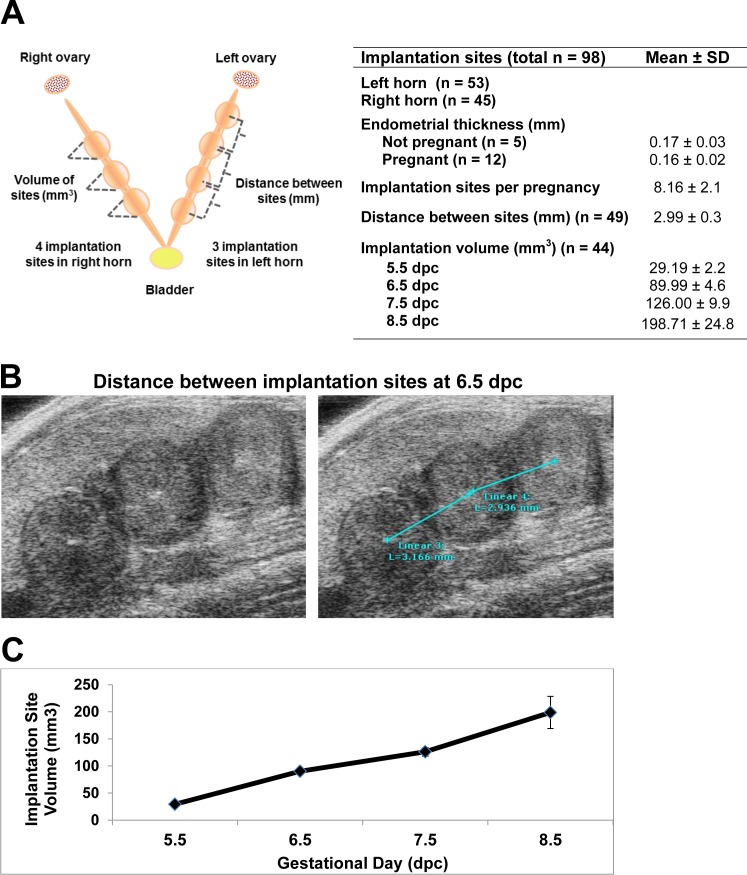
Pregnancy site growth parameters of implantation sites during early pregnancy using three-dimensional volume calculations. (**A**) As depicted in the schematic, 3-D volume reconstruction calculations allow for a graphical depiction of the size and spatial relation of each implantation size within both uterine horns. A composite of these data provide information on the distribution of the implantation sites within each horn, endometrial thickness on 0.5 d.p.c. in pregnant and non-pregnant mice, implantation sites per pregnancy, distance between sites, and implantation volume from 5.5 d.p.c. to 8.5 d.p.c. (**B**) The spacing of implantation sites is determined by the distance between each decidualized reaction, as shown. (**C**) Using the 3-D volumes at each specified day of gestation, a standardized growth curve can be developed to model a normal growth curve.

**Fig 4:**
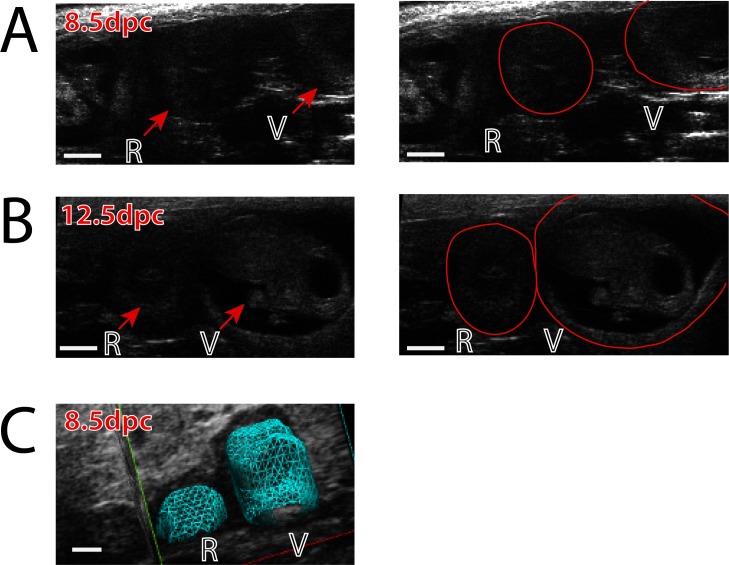
Detection and monitoring of abnormal pregnancy progression and implantation site failure using 2-D and 3-D modeling. (**A**) At 8.5 d.p.c., the resorption site of the failed pregnancy (R) is seen as compared to a neighboring viable fetus (V), with the border of each site outlined in the second image pane. (**B**) At 12.5 d.p.c., the difference between the failed resorption site (R) and viable fetus (V) is more apparent. (**C**) A 3-D visual reconstruction as early as 8.5 d.p.c. demonstrates the lagging growth of the failed resorption site (R) in relation to the adjacent viable fetus (V).

Implantation site images beginning at 5.5 d.p.c were obtained with both 2-D and 3-D imaging and correlated to gross examination of the pregnancies; representative gross examination and ultrasound images from 7.5 d.p.c. and 8.5 d.p.c are shown in **Fig 2**. High frequency ultrasound imaging was able to accurately and reliably detect the presence and location of pregnancies in each horn as confirmed by examination of the gross uterus postmortem (**Fig 2A** and **2B**). Additionally, 3-D reconstruction allowed for each pregnant uterine horn to be visually represented as a blue mesh or solid overlay that models the longitudinal perspective of the uterus with discrete implantation sites visible (**Fig 2C**). Using 3-D volume reconstruction without the surrounding contextual intra-abdominal tissue allows for the isolated uterine shape and implantation site volumes to be easily visualized in both mesh and solid overlay formats. Serial ultrasound imaging allows for the real-time depiction of the entire pregnant uterine composition, with the detection of overall implantation site number, individual embryo placement within each uterine horn, and spacing distance between each site as determined by the echogenic decidualization reaction, as shown in both pictographic and table representation in **Fig 3A**. In sum, this permits the *in vivo* determination of location of implantation sites in each horn, the average implantation sites per pregnancy, mean distance between implantation sites, and recording the increasing volume of sites beginning at 5.5 d.p.c. (**Fig 3A**). The distribution of implantation sites was equivalent between left and right horns (53 and 45 sites, respectively), with the average of 8.16 implantation sites per pregnancy. There was no difference at 0.5 d.p.c. in endometrial thickness between plugged non-pregnant and plugged pregnant mice (0.17 and 0.16 millimeters, respectively). The spacing between implantation sites at 6.5 d.p.c., as measured as distance in millimeters (mm) between decidualized sites, was 2.99 mm (**Fig 3B**). Using the 3-D reconstruction of each spherical pregnancy site allowed for the development of a standardized normal growth pattern and volume of very early pregnancy sites to be determined, increasing from 29.19 mm^3^ to 198.71 mm^3^ from 5.5 to 8.5 dpc (**Fig 3C**). Although uncommon, instances in which an implantation site stopped growing were detected, as demonstrated in **Fig 4**. As early as 8.5 d.p.c., the resorption site of the failed pregnancy is seen as compared to a neighboring viable fetus at 8.5 d.p.c (**Fig 4A**). As the gestation continues, the difference between the failed resorption site and viable fetus is more apparent (**Fig 4B**), while the 3-D reconstruction demonstrates the difference in growth as early as 8.5 d.p.c. (**Fig 4C**).

## Discussion

This short technical report describes an initial “proof-of-concept” for the use of 3-D HFUS imaging to non-invasively detect and characterize the total number of implantation sites during early murine pregnancy and to follow the developmental progression of these implantation events throughout gestation. The earliest time point of pregnancy detected in the mouse has been in a single report of ultrasound at 5.5 d.p.c., in which only 2-D ultrasound imaging was performed, without any standardized measurements, laterality, total implantation sites or implantations site spacing reported [15, 20]. Notably, many mouse models of implantation or early pregnancy failure require study in the immediate post-implantation time frame, beginning at 5.5 d.p.c. [7, 21–23]. In studies described here, the ability to accurately detect implantation site number, location, and growth from 5.5 d.p.c onwards in both 2-D and 3-D visual reconstruction in the pregnant mouse provides clear advantages over earlier studies that were limited to mid-pregnancy and used only 2-D imaging [14, 15]. Additionally, our method detects cardiac motion as soon as the embryonic mouse heart starts to beat between 8.5–9.5 d.p.c [24, 25] allowing for the detection of viable fetuses based on the initiation of cardiac activity.

For experiments in which mice are scheduled to undergo survival surgery or intervention during pregnancy, as with insertion of telemetric devices into a mid-pregnant uterus at 8.5–10.5 d.p.c. [26, 27], ultrasounds before the invasive procedure allows researchers to confirm viable pregnancies prior to subjecting the mouse to surgery. Furthermore, ultrasounds after the procedure provide reassurance that the pups have remained viable. This eliminates unnecessary experiments on non-pregnant animals, thus resulting in fewer animals required per experiment and allows for animal experimentation refinement [28–30]. Additionally, acquisition of longitudinal data in a non-invasive manner with high spatiotemporal resolution using this imaging system provides more data points per animal while reducing inter-animal variability.

High-resolution 3-D reconstruction of the uterine horn from the pregnant mouse permits individual volume measurements of each gestational sac as well as annotation of the spatial locations of pregnancy sites *in silico*. This ability to non-invasively monitor the successful (or unsuccessful) development of the fetus throughout murine gestation represents a powerful phenotyping tool to assay in real-time dynamic ultrastructural and functional changes both in the maternal and fetal environment. We anticipate that this powerful imaging system will be used to detect and monitor changes in anatomical and functional parameters *in utero* that may result from engineered genetic mutations and/or in response to endocrine or metabolic manipulation. As such, this novel technique will provide tools for researchers to further improve current investigations in various reproductive pathologies. The ability to monitor very early processes of pregnancy may detect abnormal endometrial decidualization, embryo implantation distribution and define the spatial patterning within the uterine horns. As the pregnancy progresses, non-invasive ultrasound techniques provide information regarding abnormal trophoblastic growth as well as the establishment of a functional uteroplacental unit. Overall, 2-D and 3-D high frequency ultrasound permits improved understanding of broader reproductive pathologies commonly modeled in mice, including recurrent pregnancy loss, embryonic lethality and infertility. For newly generated mouse models of early recurrent pregnancy loss or implantation failure, which are often low in number at the outset, this imaging approach would maximize the number of phenotypic data points acquired without the need to euthanize the mouse.

In conclusion, we believe that HFUS followed by 3-D reconstruction of the data *in silico* is an invaluable imaging modality for anatomical and whole uterine mapping that can be used to acquire longitudinal data in a non-invasive manner, resulting in enhanced efficiency in mouse experimentation used in the study of uterine biology and pathobiology.
